# Incidence and predictors of hypoglycemia in Japanese patients with type 2 diabetes treated by insulin glargine and oral antidiabetic drugs in real-life: ALOHA post-marketing surveillance study sub-analysis

**DOI:** 10.1186/1758-5996-6-20

**Published:** 2014-02-15

**Authors:** Masato Odawara, Takashi Kadowaki, Yusuke Naito

**Affiliations:** 1 Department of Diabetology, Metabolism, and Endocrinology, Tokyo Medical University, 6-7-1 Nishi-Shinjuku, Shinjuku-ku, Tokyo 160-0023, Japan; 2 Department of Diabetes and Metabolic Diseases, Graduate School of Medicine, The University of Tokyo, 7-3-1, Hongo, Bunkyo-ku, Tokyo 113-8655, Japan; 3 Sanofi K.K, 3-20-2 Nishi-Shinjuku, Shinjuku-ku, Tokyo 163-1488, Japan

**Keywords:** Hypoglycemia, Insulin glargine, Insulin-naïve, Oral antidiabetic drugs, Type 2 diabetes, Relative risk

## Abstract

**Background:**

**A**dd-on **L**antus® to **O**ral **H**ypoglycemic **A**gents (ALOHA), an observational, non-interventional, 24-week post-marketing surveillance study in Japanese patients with type 2 diabetes (T2DM) having uncontrolled glycemic control, demonstrated that basal supported oral therapy (BOT) with insulin glargine was an effective and safe treatment in real-life clinical practice. We performed subgroup analysis to identify incidence and predictors associated with risk of hypoglycemia.

**Methods:**

Among 4219 patients with T2DM, 3732 patients were insulin-naïve and 487 patients were insulin non-naïve who switched from other insulin to insulin glargine. All hypoglycemic episodes were counted by physicians’ documentation based on patients’ reports. Relationships between baseline patient characteristics and glargine-related hypoglycemic episodes were examined by univariate and multivariate analysis.

**Results:**

Among 4219 patients, 44 (1.0%) patients experienced hypoglycemic episodes (41 insulin-naïve patients; 3 insulin non-naïve patients), with a rate of incidence 0.035 episodes/patient-years. Majority of patients with hypoglycemia (37 of 44) had just one hypoglycemic episode during study period. Among insulin-naïve patients, incidence of hypoglycemia differed significantly depending on age, diabetic complications, estimated glomerular filtration rate (eGFR), and postprandial plasma glucose (P <0.05). In a multivariate adjusted model, poor renal function (eGFR <60 mL/min/1.73 m^2^) was a statistically significant risk factor (P < 0.05).

**Conclusion:**

Our results suggest that BOT using insulin glargine is an option of insulin therapy with 1% risk of hypoglycemia in patients with T2DM with inadequate glycemic control. Patients with low renal function might need a careful follow-up.

## Background

High prevalence of type 2 diabetes mellitus (T2DM) in Japan is associated with a significant economic burden, which increases with increasing number of diabetic complications [1]. Intensive glycemic control by multiple insulin therapy has shown to delay onset and progression of diabetic complications in Japanese patients with T2DM [2]. However, intensive glucose-lowering treatments used to achieve and maintain strict glycemic control are associated with increased risk of hypoglycemia [3-6]. Hypoglycemia impacts morbidity, mortality and quality of life of these patients [4,7-9], and also leads to higher medical expenditure [10]. Thus, it can pose a hindrance in the management of T2DM and can be major barrier in initiating and intensifying insulin treatment.

One way to ensure effective diabetes management is by opting for therapies proven to be associated with low rate of hypoglycemia. Previous data demonstrate that basal long-acting insulin analogue, insulin glargine, results in a reduced risk of nocturnal and severe hypoglycemic events compared with conventional insulin therapies [11]. Basal supported oral therapy (BOT) using insulin glargine is known to be more simple, safe and effective, as compared to neutral protamine Hagedorn (NPH) [12-15], insulin lispro [16] and premixed insulin [17,18]. Earlier studies in Japan also demonstrated that BOT with insulin glargine was effective, without causing serious hypoglycemia [19,20]. Another way to prevent or limit the incidence of hypoglycemia is to understand the underlying contributing factors. Earlier studies demonstrated that factors like old age, long disease duration, poor renal function, peripheral neuropathy, low body mass index (BMI), ≥2glucose-lowering drugs, long duration of insulin treatment, etc. are significant independent predictors of hypoglycemia [3,21,22].

The **A**dd-on **L**antus® to **O**ral **H**ypoglycemic **A**gents (ALOHA) study analyzed a large cohort of Japanese T2DM patients with inadequate glycemic control and demonstrated that BOT with insulin glargine was safe and effective [23]. It demonstrated that diabetic retinopathy, medical history, history of allergy and adverse events (AEs), and concomitant use of insulin resistance reducing agents and drugs other than OADs, were contributing factors to the occurrence of AEs. The ALOHA database yielded further sub-analysis on various aspects including dosing of insulin glargine and baseline predictive factors for achieving glycemic control that have been published [24-26].

To the best of our knowledge, there is no evidence on incidence and predictors of hypoglycemia in Japanese patients with T2DM. To address this need, we conducted a sub-analysis of the data from the ALOHA study to assess incidence of hypoglycemia and association of patient characteristics with risk of hypoglycemia in Japanese patients with T2DM. In the present sub-analysis, we stratified the ALOHA safety analysis cohort in insulin-naïve and insulin non-naive sub-groups. The present results further add to our understanding on incidence and predictors of hypoglycemia in insulin-naïve patients.

## Methods

### Study design

Between 2007 and 2009, this observational, non-interventional, 24-week follow-up, post-marketing surveillance study recruited 5223 patients from 987 centers across Japan. The detailed design and methodology of the ALOHA study is published elsewhere [23,25,26]. In the current study, Japanese patients having T2DM and inadequate glycemic control, were followed for 24 weeks to determine the incidence of hypoglycemia and any patient characteristics which predicted occurrence of hypoglycemia.

This study was endorsed by the Health Authority in Japan and was conducted as a post-marketing surveillance in accordance with the Good Post-marketing Study Practice (GPSP) [27], and Good Vigilance Practice [28] in Japan.

### Patients

Patients having T2DM who were to start BOT with insulin glargine, but who were naïve to treatment with insulin glargine, were eligible for participating in the ALOHA study. The study included patients having T2DM who satisfied the following criteria within 4 weeks screening period before initiation of insulin glargine: 1) received treatment with OADs for ≥12 weeks, 2) had HbA1c (National Glycohemoglobin Standardization Program [NGSP]*) values ≥7.9% and <12.4%, and 3) had BMI < 30 kg/m^2^.

*The NGSP values were selected based on the JDS values (≥7.5% and <12.0%, respectively). HbA1c data were collected as JDS values and then converted to NGSP values by the following conversion formula: HbA1c (NGSP) = 1.02 × HbA1c (JDS) + 0.25% with rounding off to the first decimal place [29].

### Treatment

Initiation of insulin treatment and adjusting insulin doses were determined by attending physicians. Concomitant OADs were also selected by the physicians, as part of routine clinical care.

Patients who required additional insulin, for example, bolus insulin, were terminated to follow-up due to no longer fulfilling the inclusion criteria.

### Data collection

All eligible patients’ data were collected via paper-based case report forms (CRFs). Data collected included background characteristics (gender, age, duration of diabetes, hospitalization or outpatients, disease history, complications, type and dose of prior drug therapy, etc.), treatment details, patient compliance, laboratory tests, and AEs.

### Study assessment

Safety and effectiveness data were collected over 24 weeks. Effectiveness parameters included HbA1c, fasting plasma glucose (FPG), postprandial glucose (PPG), and weight. Safety was determined by documenting all AEs and accompanying symptoms of hypoglycemia as reported by the attending physicians during the observational period. For serious AEs its seriousness, intervention, outcome and causal relationship to glargine were evaluated. Hypoglycemic episodes were counted by physicians’ documentation (any hypoglycemic episodes and any symptoms derived from hypoglycemia), based on patients’ reports. Severe hypoglycemia included hypoglycemic episodes satisfying any of the following serious AEs criteria; 1) resulted in death, 2) life-threatening, 3) required or prolonged inpatient hospitalization, 4) persistently or significantly disabling/ incapacitating, 5) a congenital anomaly, and/or 6) medically important.

### Statistical analysis

We used Fisher exact test to determine the difference between patients with or without hypoglycemia, according to patient characteristics. After calculating differences in hypoglycemia incidence and its 95% confidence intervals (CI) among subgroups, we calculated number needed to harm (NNH) by the inverse of incidence difference between subgroups. Among subgroups having statistically significant differences, we calculated multivariate adjusted relative risk estimates and 95% CI by using negative binomial regression model. All statistical tests were two-sided at α = 0.05. All statistical calculations were performed using SAS system software, version 9.1.3 (SAS Institute Inc., Cary, NC, USA) and R 2.15.2.

## Results

### Patient disposition and baseline characteristics

**Figure 1:**
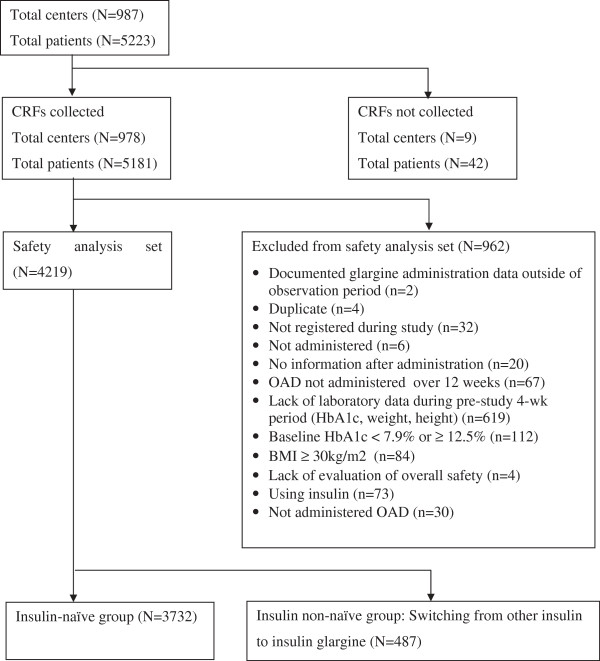
Patient disposition.

Of 5223 patients enrolled in the study, 5181 patients completed CRFs. Of these, 4219 patients were included in the safety analysis set (Figure 1). This set is further divided into 2 sub-groups: 1) Insulin-naïve group: patients treated with OADs (n = 3732), and 2) Insulin non-naïve group: patients treated with OADs + insulin other than insulin glargine, and switching to OADs + insulin glargine (n = 487).

Baseline characteristics of total and sub-group patients are presented in Table 1. Majority of patients in both groups had duration of T2DM >5 years. In insulin-naïve group receiving only OADs, majority of patients were prescribed combination of 2 or 3 OADs, while in insulin non-naïve group receiving insulin + OADs, patients commonly received 1 or 2 OADs. There were differences in the types of OADs used in insulin-naïve group (sulfonylurea [SU] – 88.9%, biguanides [BG] – 47.7%, and alpha-glucosidase inhibitors [α-GI] – 46%) and insulin non-naïve group (SU – 55.6%, α-GI – 52.2%, and BG – 42.9%). The prevalence of diabetic complications was similar in both groups.

**Table 1:** Baseline characteristics in total and sub-group patients

**Characteristics**		**Total**	Insulin-naïve group*	**Insulin non-naïve group**^**†**^
N		4219	3732	487
Male	Missing data	1	1	0
	n (%)	2485 (58.9)	2237 (59.9)	248 (50.9)
Age (years)	Missing data	8	8	0
	Mean ± SD	62.8 ± 12.1	62.6 ± 12.1	64.0 ± 12.1
Weight (kg)	Mean ± SD	61.7 ± 11.6	61.8 ± 11.7	61.0 ± 11.5
BMI (kg/m^2^)	Mean ± SD	23.8 ± 3.3	23.7 ± 3.3	24.0 ± 3.3
Duration of diabetes, years, n (%)	Missing data	241 (5.7)	221 (5.9)	20 (4.1)
	<1	50 (1.2)	46 (1.2)	4 0.8)
	≥1, <5	466 (11.0)	436 (11.7)	30 (6.2)
	≥5	3462 (82.1)	3029 (81.2)	433 (88.9)
OADs prescribed before study, n (%)	1	975 (23.1)	757 (20.3)	218 (44.8)
	2	1719 (40.7)	1537 (41.2)	182 (37.4)
	3	1189 (28.2)	1114 (29.8)	75 (15.4)
	≥4	336 (8.0)	324 (8.7)	12 (2.5)
Types of OADs prescribed before study, n (%)	BG	1991 (47.2)	1782 (47.7)	209 (42.9)
	SU	3587 (85.0)	3316 (88.9)	271 (55.6)
	Glinides	312 (7.4)	269 (7.2)	43 (8.8)
	α-GI	1971 (46.7)	1717 (46.0)	254 (52.2)
	TZD	1360 (32.2)	1287 (34.5)	73 (15.0)
Diabetic complications	Neuropathy	1083 (25.7)	933 (25.0)	150 (30.8)
	Retinopathy	1148 (27.2)	971 (26.0)	177 (36.3)
	Nephropathy	1121 (26.6)	953 (25.5)	168 (34.5)
eGFR (mL/min/1.73 m^2^)	Missing data	997 (23.6)	892 (23.9)	105 (21.6)
	≥90	1030 (24.4)	933 (25.0)	97 (19.9)
	≥60, <90	1558 (36.9)	1375 (36.8)	183 (37.6)
	<60	634 (15.0)	532 (14.3)	102 (20.9)

Note:

*Insulin-naïve group: patients having been treated with OADs (n = 3732).

^**†**^Insulin non-naïve group: patients treated with OADs + insulin other than insulin glargine, and switching to OADs + insulin glargine (n = 487).

*Abbreviations*: *α-GI* alpha-glucosidase inhibitors, *BG* biguanide, *BMI* body mass index, *eGFR* estimated glomerular filtration rate, *OADs* oral antidiabetic drugs, *SD* standard deviation, *SU* sulfonylurea, *TZD*, thiazolidinedione.

The HbA1c value at baseline in the naïve population was 9.53 ± 1.19% (n = 3732). In the non-naïve population the baseline HbA1c value was 9.08 ± 1.11% (n = 487). The HbA1c values at the end of the study were 8.07 ± 1.21% (n = 3337) and 8.46 ± 1.39% (n = 424) in the naïve and non-naïve populations, respectively.

### Hypoglycemia

**Figure 2:**
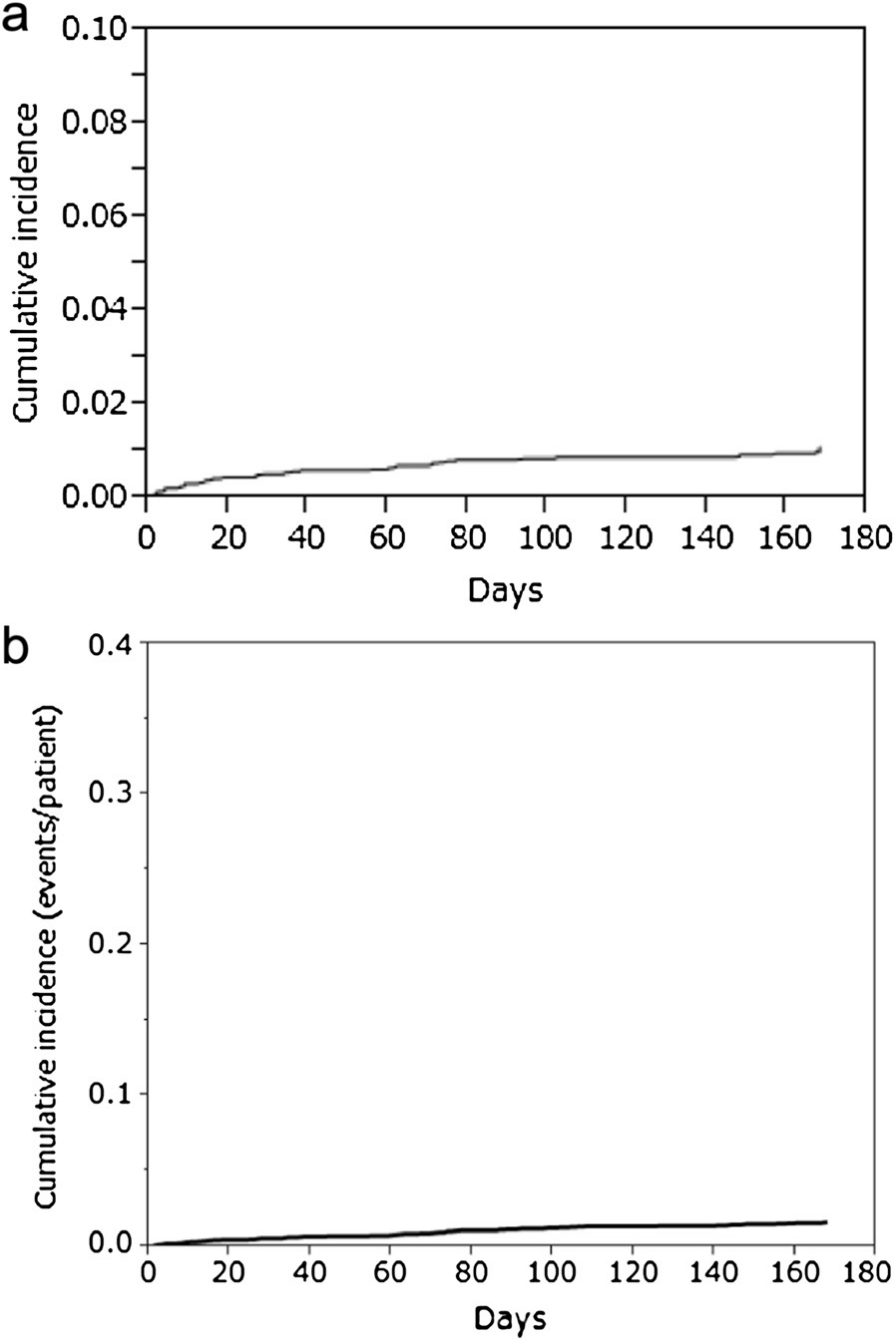
a) Cumulative incidence and b) cumulative incidence rate (episodes/patient) of hypoglycemia.

The data on incidence of hypoglycemia in total as well as subgroup patients have been presented in Table 2. In total patients, incidence of hypoglycemia was 1.0% (0.035 episodes/patient-years). Incidence of hypoglycemia in insulin-naïve group (1.1% [0.036 episodes/patient-years]) and insulin non-naïve group (0.6% [0.029 episodes/patient-years]) was similar to the overall incidence. The difference in the incidence of hypoglycemia between naïve and non-naïve groups was not statistically significant; p-value was 0.4752 by fisher exact test. Among total 4219 patients, 4 (0.1%) patients had severe hypoglycemia (0.002 episodes/patient-years). At the end of the 24-weeks study period, cumulative incidence of hypoglycemia was 1.0% (Figure 2a), and cumulative incidence rate of hypoglycemia was 0.016 episodes/patient (Figure 2b).

**Table 2:** Cumulative incidence and rate of hypoglycemia in overall and sub-group patients

**Parameters**	**Total**	Insulin-naïve group*	**Insulin non-naïve group**^**†**^
N	4219	3732	487
Patients with hypoglycemia, n (%)	44 (1.0)	41 (1.1)	3 (0.6)
Patient-years	1801.2	1596.7	204.5
Episodes, n	63	57	6
Incidence rate (episodes/patient-year)	0.035	0.036	0.029

Note:

*Insulin-naïve group: patients having been treated with OADs (n = 3732).

^**†**^Insulin-naïve group: patients treated with OADs + insulin other than insulin glargine, and switching to OADs + insulin glargine (n = 487).

Altogether, 44 patients reported 63 hypoglycemic episodes: 37 (0.88%) patients had 1 episode, 3 (0.07%) had 2 episodes, 2 (0.05%) had 4 episodes, and 1 (0.02%) patient each had 5 and 7 episodes. The details of the 63 hypoglycemic episodes were as follows: 20 episodes with convincing symptoms, 31 episodes in patients with morning injection, 24 episodes in patients with bedtime injection, 4 episodes identified by self-monitored blood glucose measurement (2 patients), and 2 episodes in the night (1 in evening and 1 with bedtime injection).

### Hypoglycemia incidence and rate according to patient characteristics in insulin-naïve group: univariate analysis

When we assessed incidence of hypoglycemia and its rate with various patient characteristics in insulin-naïve patients, there was no statistically significant difference in sub-groups such as sex, BMI, duration of diabetes, renal disorder not due to diabetes, baseline HbA1c, FPG, OADs prescribed prior to and during study, and compliance to diet and exercise (data not shown). There was a statistically significant difference in hypoglycemic incidence in insulin-naïve patients in subgroups according to age, diabetic complications, estimated glomerular filtration rate (eGFR), and PPG levels (Table 3). Among the diabetic complications, the NNH was low for retinopathy and/or nephropathy. Also, for eGFR <60 mL/min/1.73 m^2^, the NNH was lower than eGFR levels of >60 mL/min/1.73 m^2^.

**Table 3:** Cumulative incidence and rate of hypoglycemia in insulin-naïve group (N = 3732), according to patient characteristics

**Characteristics**		**Total patients, N**	**Patients having hypoglycemia, n (%)**	**P value**	**Number needed to harm (95% CI)**	**Patient-years**	**Episodes, n**	**Incidence rate (episodes/patient-year)**
Age (years)	<65	2046	14 (0.7)	< 0.05	Reference	880.5	21	0.024
	≥65	1678	27 (1.6)		108 (62–445)	714.2	36	0.050
Diabetic complications	No microvascular complication	1889	18 (1.0)	< 0.05	Reference	811.1	24	0.030
	Neuropathy only	297	6 (2.0)		94 (n.s.)	127.9	6	0.047
	Retinopathy only	318	1 (0.3)		-*	138.3	1	0.007
	Nephropathy only	356	2 (0.6)		-*	152.5	5	0.033
	Neuropathy + Retinopathy	174	1 (0.6)		-*	74.9	1	0.013
	Neuropathy + Nephropathy	142	0 (0.0)		-*	59.5	0	0.000
	Retinopathy + Nephropathy	154	5 (3.2)		44 (n.s.)	64.1	7	0.109
	Neuropathy + Retinopathy + Nephropathy	301	5 (1.7)		141 (n.s.)	126.4	6	0.047
eGFR (mL/min/1.73 m^2^)	90≤	933	3 (0.3)	< 0.01	Reference	400.6	4	0.010
	60≤, <90	1375	12 (0.9)		181 (n.s.)	589.0	13	0.022
	<60	532	11 (2.1)		57 (33–207)	225.0	16	0.071
	Unknown	892	15 (1.7)		74 (44–227)	382.1	24	0.063
PPG (mg/dL)	<140	45	1 (2.2)	< 0.01	57 (n.s.)	19.1	1	0.052
	140≤, <180	132	5 (3.8)		30 (15–4008)	57.2	8	0.140
	180≤, <220	235	3 (1.3)		125 (n.s.)	103.5	7	0.068
	≥220	1043	5 (0.5)		Reference	446.7	13	0.029

Note: Since the incidence of hypoglycemia was very low in insulin non-naïve group patients (3 patients having 6 episodes of hypoglycemia), we have not reported the univariate analysis of that data.

Insulin-naïve group: patients having been treated with OADs (n = 3732).

Insulin non-naïve group: patients treated with OADs + insulin other than insulin glargine, and switching to OADs + insulin glargine (n = 487).

Each of the subgroups were compared to 'Reference’ subgroup by all characteristics.

*Abbreviations*: *CI* confidence interval, *eGFR* estimated glomerular filtration rate, *PPG* post-prandial glucose, *n.s* not significant.

*These were not estimated because incidence rates were below that of the reference category.

Since, there were only 6 episodes of hypoglycemia in 3 patients in insulin non-naïve group, we did not assess them in the univariate analysis.

### Predictors of hypoglycemia in insulin-naïve group: multivariate analysis

All factors having a statistically significant difference in the univariate analysis (Table 3), were examined by multivariate analysis. PPG was excluded because of missing data. Due to a very few hypoglycemic episodes, diabetic complication category was forced to be contracted into dichotomous unlike all combinations of complications presented in Table 3. Multivariate adjusted negative binomial regression model revealed that among the parameters studied, poor renal function defined as eGFR <60 mL/min/1.73 m^2^ was the only statistically significant risk factor of hypoglycemic events (relative risk [RR] 5.34, 95% confidence interval [CI] 1.48-22.85, P <0.05).

## Discussion

This observational, non-interventional, 24-week post-marketing surveillance study in Japan provides detailed real-life information on incidence of hypoglycemia in T2DM patients using BOT with insulin glargine. The overall incidence of hypoglycemia was 1.0%, with majority of patients experienced hypoglycemia only once during 24-week follow-up period. The study also demonstrated, for the first time, that among insulin-naïve T2DM Japanese patients, hypoglycemia is associated with poor renal function.

The prospective, observational registry in Germany demonstrated that hypoglycemia is a frequent AE in insulin-naïve T2DM patients having insufficient glycemic control on OADs, and receiving intensified antidiabetic treatment [30]. The rate of hypoglycemia in this study, over a 12-month follow-up period, was mild: 13.0%, moderate: 0.7%, and severe: 0.5%. The results of this study indicated that the risk of hypoglycemia might be substantially reduced by carefully selecting antidiabetic pharmacotherapy [31]. Earlier randomized trials using BOT with insulin glargine reported high incidence of hypoglycemia. In the HOE 901/3002 study, 33% of insulin-naïve patients receiving insulin glargine experienced at least one episode of symptomatic hypoglycemia, and 9.9% patients experienced nocturnal hypoglycemia [12]. In the HOE 901/2004 study, among T2DM patients having inadequate glycemic control on OADs, 22.1% and 7.3% of patients receiving insulin glargine experienced symptomatic hypoglycemia and nocturnal hypoglycemia, respectively [13]. Other studies reported the hypoglycemia incidence rate in the range of 0.7 to 5.2 episodes per patient-year [14,16,17,32]. Outside the clinical setting, an earlier observational study reported 0.1% prevalence of hypoglycemia [33]. In the current study, rate of hypoglycemia in insulin-naïve patients was 1.1%, which is higher than that reported in the earlier observational study. The difference in the rate of hypoglycemia can be due to heterogeneity of the study populations and different definitions of hypoglycemia used in these studies. The higher hypoglycemia rate in clinical trials could be due to intensive antidiabetic treatments used to achieve glycemic targets, as opposed to the observational studies.

The American Diabetes Association/European Association for the Study of Diabetes (ADA/EASD) guidelines [34] and the Japan Diabetes Society (JDS) treatment guide [35] recommend intervention at the time of diagnosis, with OADs in combination with lifestyle changes in diet and exercise. In patients who do not meet glycemic goals by taking only OADs, the guidelines recommend timely augmentation of this therapy with additional agents and early addition of insulin therapy. However, there exist patient barriers such as fear of hypoglycemia, injections and weight gain, as well as physicians’ concerns such as reluctance to prescribe insulin, result in non-adherence to initiation and intensification of insulin treatment, leading to delayed use of effective therapy [36,37]. Early initiation of insulin therapy might help patients with T2DM achieve long-term glycemic control and improve quality of life. It has been demonstrated that first insulinization with basal insulin is effective and safe with reduced AEs including hypoglycemia, in clinical trial [38], as well as real-world setting [15]. However, in the current study, majority of patients had T2DM for more than 5 years and still were prescribed only one and/or two OADs (61.5%) and were insulin-naïve (81%). Thus, physician and patient education is necessary to overcome barriers to insulin use and ensure its appropriate and optimal use.

In the present study, we demonstrated association of hypoglycemia with older age (relative risk [RR] 1.58, 95% CI: 0.74, 3.44). Advanced age has been a contributing factor to severe hypoglycemia in previous population-based studies [39,40]. Earlier ACCORD [41] and ADVANCE [9] trials reported significant associations between older age and risk of severe hypoglycemia. In an earlier study in insulin-naïve T2DM patients, age <65 years was an independent predictor of reduced incidence of hypoglycemia (OR 0.76; 95% CI 0.59-0.96) [31]. The DAWN Japan study demonstrated that old age was one of the top three concerns for physicians to delay insulin initiation [42]. Hence, future studies to address appropriate strategies to overcome these physician barriers are warranted.

In ADVANCE study, history of microvascular disease was associated with twofold increase in risk of severe hypoglycemia (hazards ratio 2.1, 95% CI 1.5 to 3.) [9]. Diabetic complications such as coronary artery disease and peripheral neuropathy are also known as risk factors for hypoglycemia [22,43]. In line with the earlier evidence, in the current study also, hypoglycemia was associated with presence of diabetic complication (s) (RR 1.36, 95% CI: 0.66, 2.83). Many earlier studies reported poor renal function diagnosed by low eGFR as a significant predictor of first of recurrent hypoglycemia [22,43]. Our results confirm this by showing significant association of hypoglycemia and poor renal function i.e. eGFR <60 mL/min/1.73 m2 (RR 5.34, 95% CI: 1.48, 22.85). However, the risk difference was very low and not clinically relevant.

One of the inclusion criteria of the current study is BMI <30 kg/m^2^. Accordingly, the mean BMI of the patients in safety analysis set was 23.8 kg/m^2^. Earlier studies demonstrated that low BMI was an independent risk factor of severe hypoglycemia [9,41]. However, these studies recruited global population of T2DM patients. In the current study, the incidence of severe hypoglycemia was 0.1%. This can be due to the lower cut-off levels of BMI for overweight and obesity in Asian population as compared to the Western population [44].

The results of the present observational study explore the incidence and predictors of hypoglycemia in real-life clinical practice in insulin-naïve diabetic patients in Japan having inadequate glycemic control and who are starting with BOT with insulin glargine. However, this population might not represent general T2DM patient population who initiate insulin glargine. Also, unlike a treat-to-target trial, the present observational study has no interventional target of glycemic control. Another limitation of the study is that because of the observational nature of the study, it is likely that incidence of hypoglycemia might have been under-reported. The data on hypoglycemia in patients receiving insulin other than glargine and switching to insulin glargine will be further elaborated in subsequent publication, which would include comprehensive results of effectiveness and safety in this cohort.

## Conclusions

In conclusion, our results suggest that BOT using insulin glargine is an option of insulin therapy with a 1% risk of hypoglycemia in insulin-naïve patients with T2DM with inadequate glycemic control. Age, presence of diabetic complications, and poor renal function were the predictors of hypoglycemia in insulin-naïve patients. Patients with low renal function might need a careful follow-up.

## Abbreviations

α-GI: Alpha-glucosidase inhibitors; ADA: American diabetes association; AE: Adverse event; EASD: European association for the study of diabetes; BG: Biguanide; BMI: Body mass index; BOT: Basal supported oral therapy; CI: Confidence interval; CRF: Case report form; GPSP: Good Post-marketing Study Practice; eGFR: Estimated glomerular filtration rate; FPG: Fasting plasma glucose; HbA1c: Glycosylated hemoglobin; JDS: Japan diabetes society; NNH: Number needed to harm; NPH: Neutral protamine Hagedorn; OAD: Oral antidiabetic drug, PPG, prandial plasma glucose; RR: Relative risk; SU: Sulfonylurea; T2DM: Type 2 diabetes mellitus; TZD: Thiazolidinedione.

## Competing interests

MO received advisory board fees, lecture fees, honoraria for manuscripts, and scholarship grants fees from Sanofi K.K. TK received advisory board fees, lecture fees and scholarship grants fees from Sanofi K.K.YN is an employee of Sanofi K.K.

## Authors’ contributions

Sanofi was responsible to design and conduct this study. YN was responsible for the statistical analysis. MO and TK made significant suggestions to the analysis and interpretation of data. YN drafted the manuscript, MO and TK reviewed and revised the draft manuscript. All authors have reviewed and approved the final version of this manuscript.
